# Supplementary material for: Aurora A inhibition limits centrosome clustering and promotes mitotic catastrophe in cells with supernumerary centrosomes
Source: Oncotarget. 2019 Feb 26;10(17):1649–59. doi: 10.18632/oncotarget.26714 (PMC6422193; doi:10.18632/oncotarget.26714)
Supplement: Supplementary file 1 [file oncotarget-10-1649-s001.pdf]

Aurora A inhibition limits centrosome clustering and promotes mitotic catastrophe in cells with supernumerary centrosomes

SUPPLEMENTARY MATERIALS

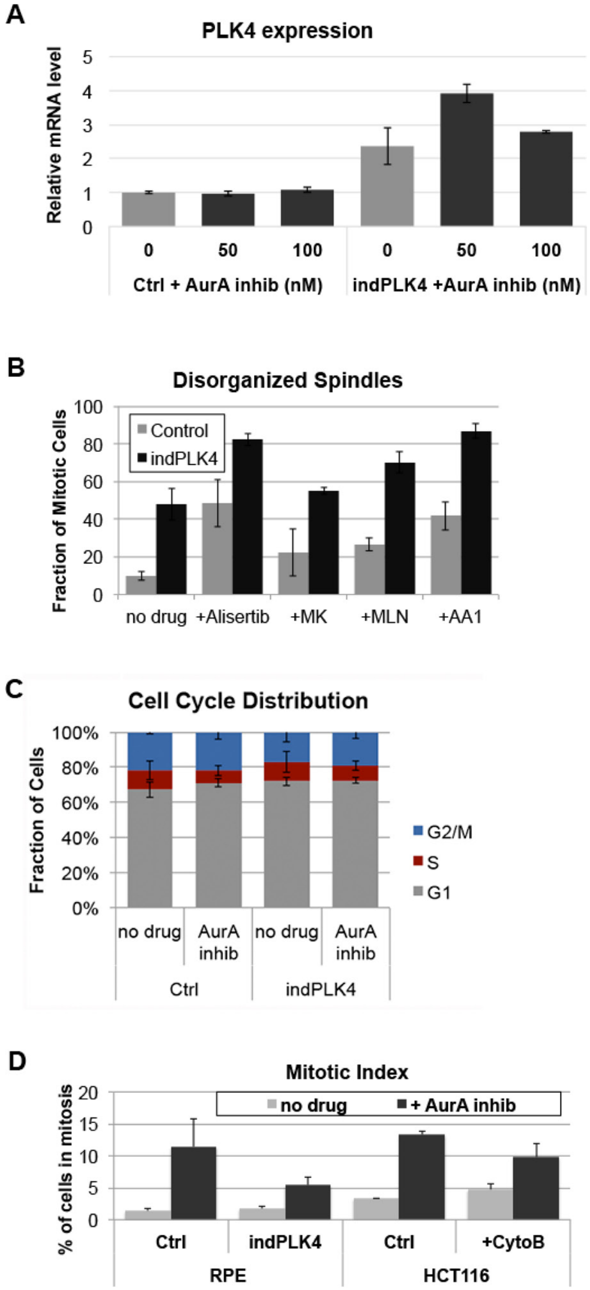

Supplementary Figure 1: Short-term inhibition of Aurora A disrupts spindle structure without altering overall cell cycle distribution. (**A**) PLK4 expression is efficiently induced for 36 h from a doxycycline-regulated expression construct in RPE-1 cells to promote centriole over-duplication (see also Figure 1). (**B**) A panel of four clinically relevant, Aurora A inhibitors (alisertib; MLN8054, Aurora A inhibitor 1, and MK-5108 (VX-689)) all similarly corrupted spindle structure in RPE-1 cells with or without supernumerary centrosomes (indPLK4). (**C** and **D**) Inhibition of Aurora A kinase activity with 100 nM alisertib for 16 h does not alter cell cycle distribution as judged by FACs analysis of DNA content, and only moderately increases the fraction of cells observed to be in mitosis by immunofluorescence based assay.

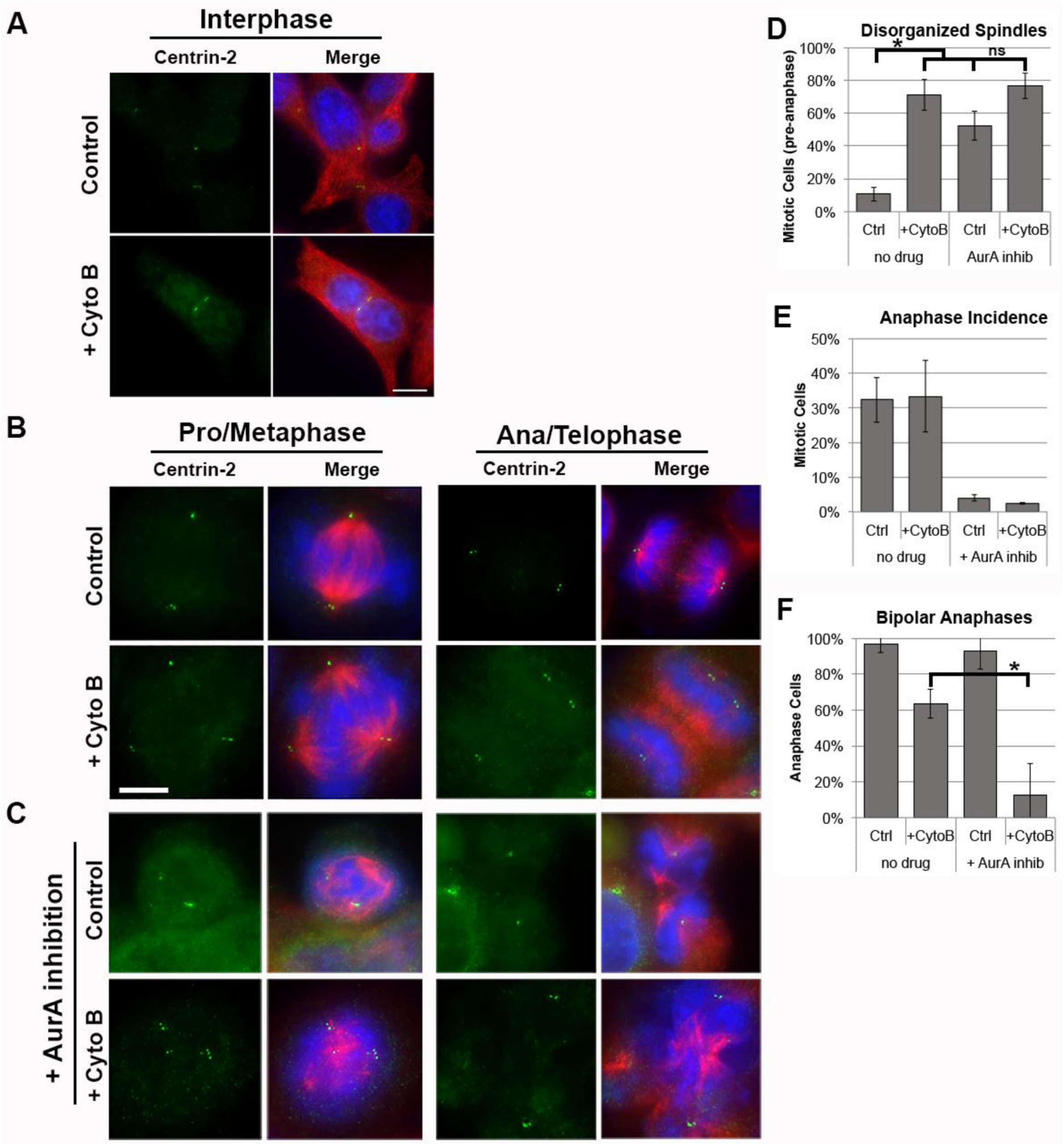

Supplementary Figure 2: Inhibition of supernumerary centrosomes clustering is a general result of Aurora A inhibition and not specific to treatment with alisertib. (**A**) Treatment of HCT116 p53−/− cells with cytochalasin B (Cyto B) to induce cytokinesis failure results in binucleated cells with extra centrosomes. (**B**) Similar to indPLK4 expressing RPE cells, HCT116 cells treated with Cyto B are able to efficiently cluster extra centrosomes to achieve a bipolar spindle prior to anaphase. (C and D) HCT116 cells with and without extra centrosomes form multipolar spindles when Aurora A is inhibited. (**C–F**) In the presence of AurA inhibition, cells with 2 centrosomes are able to achieve spindle bipolarity prior to anaphase onset, while cells with extra centrosomes primarily exit mitosis with multipolar spindles.

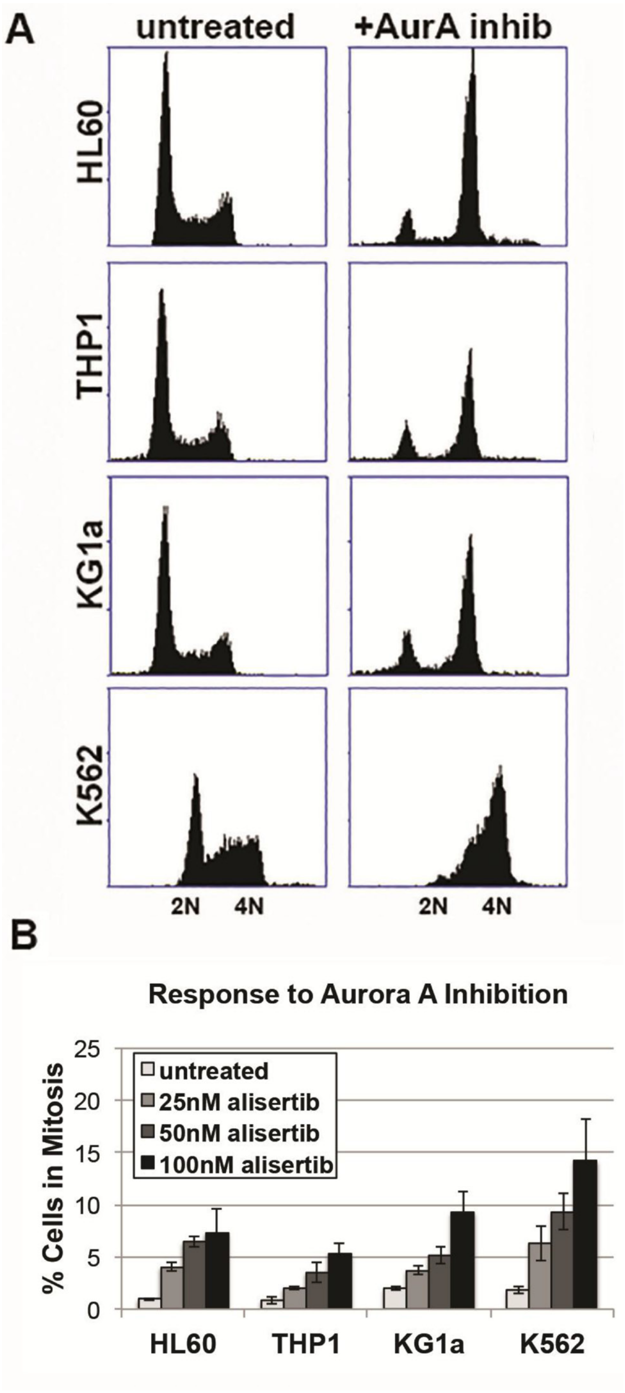

Supplementary Figure 3: Cell cycle response to Aurora A inhibition is similar in AML cells with two or supernumerary centrosomes. (**A**) FACs analysis indicates that all AML cell lines exhibit an initial enrichment in cells with 4N (G2/M) DNA content following short term exposure to the Aurora A inhibitor alisertib. (**B**) Immunofluorescence analysis of mitotic figures shows that Aurora A inhibition induces a concentration-dependent enrichment in mitotic cells similarly in all four of these cell lines, while only promoting multipolar spindle structures in the cell line with supernumerary centrosomes (see also Figure 4F).

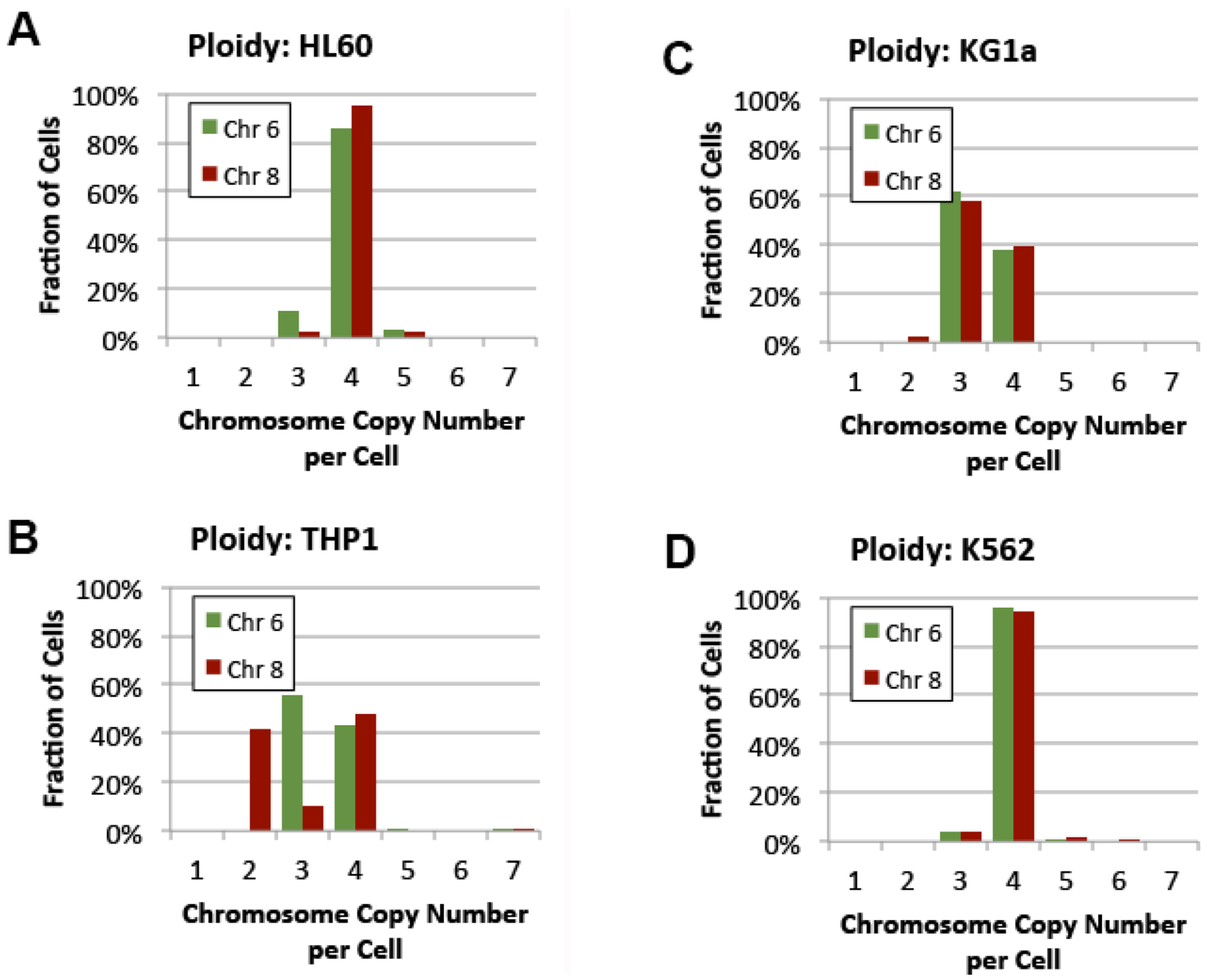

Supplementary Figure 4: Ploidy does not correspond with centrosome number in AML cell lines. FISH-based analysis of copy number for chromosomes 6 (green) and 8 (red) indicate that each of the 4 AML cell lines investigated had a modal chromosome copy number near 4, while only K562 cells exhibited supernumerary centrosomes (see Figure 4), indicating that ploidy alone is not a strong indicator of centrosome number.
